# Chicago Pathological Society

**Published:** 1883-06

**Authors:** H. M. Lyman

**Affiliations:** President


### ARTICLE VII.

CHICAGO PATHOLOGICAL SOCIETY. Stated Meeting, Dec. 11th, 1882. Dr. H. M. Lyman, President, in the Chair.

Dr. G. Frank Lydston, late resident surgeon to Charity and State emigration hospitals, New York, and now lecturer on genito-urinary disease in the College of Physicians and Surgeons of Chicago, read a paper entitled, "A Contribution to the Hereditary and Pathological Aspect of Vice," which will be found in the February number of the JOURNAL. The society had turned out in full, and several of the members took place in the following discussion.

### DISCUSSION.

Prof. E. L. Holmes said that this was certainly a great subject. He had been brought in contact with a large number of criminals, and it was evident that just as we come across cases of color-blindness, we similarly meet cases of moral blindness. The organization might be faultless otherwise, still there was in many criminals a deficient appreciation of the morality of certain acts, and that deficiency seemed rooted in their constitution. He related the instance of a banker who never could take a liking to anything. He cited instances of perversions of taste, and looked upon this conjointly with color-blindness as next to incurable, and he thought this applied to a large class of criminals. In our incapacity to abolish crime we had to bear with it.

Prof. J. J. M. Angear, of the College of Physicians and Surgeons, who was present by invitation, was asked to take part in the discussion, and made the following remarks: He had had a large experience in the treatment of criminals in the capacity of surgeon to a penitentiary for many years. He denied the inferiority of mental power ascribed to that class by so many writers. He had often been surprised at the proficiency of many of them in languages, the scriptures, etc. He understood that the sulci of the brain were evidences of an increase of the gray matter and consequently represented an increase of force generally in the individual. In cases of this kind, if the nature or will of the individual became depraved, the greater would the criminal be. He believed in intellectual criminals, that is in criminals who kept abreast of the progress of civilization. Thus, since the common poisons were so readily detected, some criminals would select the rarer ones; after the microscope had revealed the fact that the blood of a dog resembled that of man some murderers would call the blood on their clothes dog's blood, etc. Although the inheritance of pauperism had not been brought up before the society, it was one of the most manifest instances of heredity. Some wealthy people, suddenly deprived of all their means, generally took but a short time to rise again, while families of paupers had often been continued for innumerable generations. As to mental impressions made by the mother on the fœtus, he thought there was a little of truth in it, but heredity mostly depended on the impressions made on the spermatozoa and ova while they were in process of formation in the parents. And reformations were not so liable to be transmitted to the progeny as were the results of early education in the parents. Hence, we should bear in mind that young girls and boys in the early years of their life were forming the minds of the coming generation, and that their education on that account should on that account receive greater care. He related instances of rabid temperance men, born of a mother who abhorred liquor, and of a drunkard father, to show that inheritance might sometimes be one-sided. As to prostitution, he believed that it was probably often transmitted from father to daughter, and that its burden should not rest on the female alone.

Dr. Lewis said that he was not entirely satisfied with the treatment of the subject under consideration, because it had not been explained why there was in certain individuals a lack of moral sense which existed in others, or why should an inferior cerebral development exist in some individuals.

Dr. Odelia Blinn talked at some length on the subject of prostitution, with special reference to a case now before the grand jury. She said she did not believe in calling prostitution a sin as long as it was looked upon as such only by men in their appreciation of women. What was legitimate in one could not be a sin in the other.

After a few more physicians had spoken on the subject, Prof. H. M. Lyman said that he was well aware of the great difference which existed between various intellects, and quoted the statement of a celebrated writer to the effect that there was a greater difference between the higher and lower races of man than between these and the lower animals. Yet, this difference was not one of configuration, but one which should be looked for in the brain mostly. Criminals had certain perceptions of physical relations, but it was probable that they did not have the power of seeing certain moral relations, and in this must consist the greatest difference between men. There was a general resemblance in brains, corresponding to a general sense of morality in mankind. The only remedy applicable to vices, besides repressive law, should consist in making up for a deficient moral sense by proper moral education. Children should be trained into morality. This did not mean religious, dogmatic or theological teaching, but the inculcation of a moral habit. It was a grave error to confine education in the public school to intellectual training only, and a reform was needed in that direction.

---

EXPLOSIVE MIXTURES OF MEDICINE.—SEMINARIO FARMACEUTICO.

1. Hypophosphite of lime, chlorate of potash and sulphate of iron, mixed in equal proportions, are explosive.

2. A solution of one part of chromic acid and two of glycerine.

3. Chlorate of potash and dental powders containing carbon explode in the mouth.

4. A pilular mass containing permanganate of potash, mixed with vegetable extracts and iron, easily inflames.

5. Chlorate of potash or the permanganate or the other explosive substances must not be triturated with glycerine.—*Rev. Scienc. Med.—Rev. Md. Quirurgica.*

Chlorate of potash and tannin explode if triturated, as do chlorate of potash and sugar. Iodine or an iodide and a nitreta may explode.—*Virginia Med. Monthly.*

